# MET 14外显子跳跃突变在非小细胞肺癌中的研究进展

**DOI:** 10.3779/j.issn.1009-3419.2018.07.09

**Published:** 2018-07-20

**Authors:** 利梅 尹, 铀 卢

**Affiliations:** 610041 成都，四川大学华西医院胸部肿瘤科 Department of Thoracic Oncology, West China Hospital, Sichuan University, Chengdu 610041, China

**Keywords:** 肺肿瘤, MET, 酪氨酸激酶抑制剂, 克唑替尼, 靶向治疗, Lung neoplasms, MET, Tyrosine kinase inhibitors, Crizotinib, Targeted therapy

## Abstract

近年来，靶向治疗在非小细胞肺癌（non-small cell lung cancer, NSCLC）患者的治疗中取得了巨大成功。间质-上皮细胞转化因子（mesenchymal-epithelial transition factor, MET）被认为是继表皮生长因子受体（epidermal growth factor receptor, EGFR）、间变性淋巴瘤激酶（anaplastic lymphoma kinase, ALK）之后又一重要的NSCLC分子治疗靶点。MET 14外显子跳跃突变患者在一些临床试验及个案报道中显示出对MET抑制剂良好的疗效，提示MET 14外显子跳跃突变或可成为靶向治疗的良好指标，但这仍需大样本量的临床研究来证实。本文就MET 14外显子跳跃突变的分子机制、人群特征、治疗策略及耐药机制作一综述。

## Abstract (en)

Recently, targeted therapy has achieved great success in the treatment of non-small cell lung cancer (NSCLC) patients. Mesenchymal to epithelial transition factor (MET) is considered to be another important molecular target for NSCLC since epidermal growth factor receptor (EGFR) and anaplastic lymphoma kinase (ALK). Accumulating clinical trials and case reports have confirmed that MET inhibitors exhibited a potential prospect in treating patients with MET 14 exon skipping alterations, suggesting that MET 14 exon skipping mutation might be an effective biomarker for MET inhibitors, which remains to be confirmed by more clinical data. This review summarizes current research about the molecular mechanism, clinicopathological characterization, treatment strategies and drug resistance mechanisms of MET 14 exon skipping alterations in NSCLC.

尽管目前肺癌仍然是世界上致死率最高的肿瘤，但在具有某些特定基因突变的非小细胞肺癌（non-small cell lung cancer, NSCLC）患者中，靶向治疗的出现使患者的预后有了较大的提升^[1, 2]^。间质-上皮细胞转化因子（mesenchymal-epithelial transition factor, *MET*）与表皮生长因子受体（epidermal growth factor receptor, *EGFR*），鼠类肉瘤病毒癌基因（kirsten rat sarcoma viral oncogene, *KRAS*）等癌基因近乎同时被发现（1984年）^[3]^。然而与同时代分子靶点的瞩目成果不同的是，尽管目前已经研制出超过20种靶向MET及其配体肝细胞生长因子（hepatocyte growth factor, HGF）的药物，数个Ⅲ期临床试验均以失败告终^[4, 5]^。近年来，越来越多的证据表明MET抑制剂在MET 14外显子跳跃突变的患者中取得了良好的抗肿瘤效果，提示MET 14外显子跳跃突变或可作为NSCLC患者治疗的新靶点^[6]^。本文就MET 14外显子跳跃突变的分子机制、人群特征、治疗策略及耐药机制进行综述。

## 1 MET 14外显子跳跃突变的分子机制

作为一种跨膜酪氨酸激酶受体，MET与配体HGF结合后，发生二聚化并引起细胞内多种酪氨酸残基的磷酸化，进而激活一系列下游信号通路，包括Ras-MAPK、PI3K-Akt等，进而发挥其促细胞增殖、生长、迁移及血管生成等效应。与其他酪氨酸激酶受体一样，MET由E3泛素连接酶c-Cbl降解。MET 14外显子编码的近膜结构域是MET的关键负性调控区，包含一段半胱天冬酶裂解序列和一个E3泛素连接酶c-Cbl酪氨酸结合位点（Y1003），参与MET蛋白的泛素化和降解。MET 14外显子剪接供体及受体位点的突变会引起外显子跳读（exon skipping），含有E3泛素连接酶c-Cbl的近膜结构域缺失，进而使MET蛋白泛素化障碍及降解率减低，增加MET的稳定性，引起下游信号的持续激活，最终成为致癌因子^[7]^（图 1）。目前研究报道的MET 14外显子跳跃突变形式多种多样，包括MET 14外显子剪接区域的点突变或缺失突变，以及极少数Y1003点突变^[8]^。

**1:**
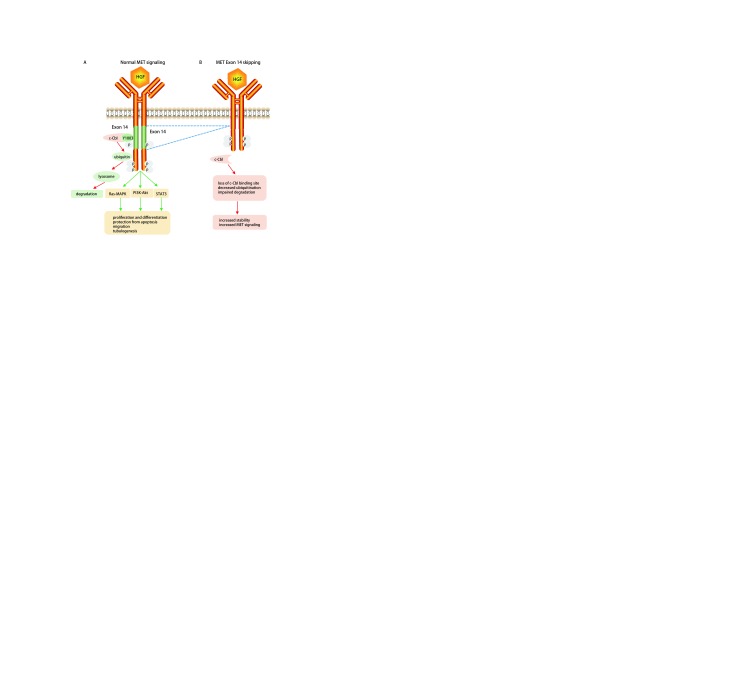
MET 14外显子跳跃突变的分子机制。 The molecular mechanism of MET exon 14 skipping.

尽管MET 14外显子跳跃突变早在20年前就已被发现，但直到2014年7月，美国癌症基因研究组（The Cancer Genome Atlas, TCGA）通过对230例肺腺癌的mRNA和DNA高通量测序结果进行序列比对分析，发现约4%（10/230）的肺腺癌存在MET 14外显子跳跃突变，才使研究者开始关注这一基因靶点（表 1）^[9]^。

**1:** MET 14外显子突变患者的临床研究 Clinical studies of MET exon 14 skipping alterations

Year	Country	Histology included	Diagnostic technology	Concurrent MET amplication	Incidence	Reference
2014	USA	Adenocarcinoma	WES	NR	4.3% (10/230)	TCGA^[9]^
2015	USA	All histologies	Hybrid capture NGS	NR	3% (131/4, 402) in adenocarcinoma; 2.3% in other lung histologies	Frampton, *et al*^[15]^
2016	USA	NSCLC	NGS	21%	3% (28/933) in non-squamous NSCLC; 0% in squamous cell carcinoma	Awad, *et al*^[10]^
2016	China	NSCLC	PCR, Sanger sequencing	33.3%	2.6% (10/392) in adenocarcinoma; 4.8% in adenosquamous cell carcinoma; 31.8% in pulmonary sarcomatoid carcinoma; 0% in squamous carcinoma; 0% in large cell carcinoma	Tong, *et al*^[12]^
2016	USA	NSCLC	Anchored multiplex RNA sequencing	6.3% (1/16)	5.6% (5/89)	Heist, *et al*^[16]^
2016	USA	Pulmonary sarcomatoid carcinoma	WES, RT-PCR	NR	22.2% (8/36)	Liu, *et al*^[17]^
2016	China	NSCLC	NGS, Sanger sequencing	No	0.9% (10/1, 101) in adenocarcinoma; 5% in squamous cell carcinoma; 0.7% in adenosquamous cell carcinoma	Liu, *et al*^[18]^
2016	USA	All lung cancer histologies	Hybrid capture NGS	14.8%	2.7% (298/11, 205) in all histologies; 8.2% in adenosquamous cell carcinoma; 7.7% in sarcomatoid carcinoma; 2.9% in adenocarcinoma; 2.1% in squamous cell carcinoma; 0.8% in large cell carcinoma; 0.2% in SCLC	Schrock, *et al*^[20]^
2017	Korea	Adenocarcinoma and pleomorphic carcinoma	qRT-PCR	NR	8.8% (9/102) in triple-negative adenocarcinoma; 20% in pleomorphic carcinoma	Kwon, *et al*^[19]^
2017	Taiwan	All lung cancer histologies	one-step RT-PCR	NR	3.3% (28/850) in all histologies; 4% in adenocarcinoma	Gow, *et al*^[11]^
2017	Korea	NSCLC	RT-PCR	NR	2.1% (17/795) in NSCLC; 37.8% in quintuple-negative lung adenocarcinoma	Lee, *et al*^[13]^
WES: whole-exome sequencing; NSCLC: non-small cell lung cancer; NR: not reported; NGS: next generation sequencing; RT-PCR: reverse transcription-polymerase chain reaction.						

## 2 MET 14外显子跳跃突变患者的临床病理特征

2016年，Award等^[10]^利用二代测序的方法检测了933例NSCLC患者的基因，发现28例NSCLC患者中存在MET 14外显子跳跃突变，约占3.0%，并且这28例患者中均没有*EGFR*或*KRAS*突变。后续有研究^[11, 12]^发现MET 14外显子突变患者均未发现*ALK*或*ROS1*基因重排。在其他驱动基因（*EGFR*/*KRAS*/*ALK*/*ROS*/*RET*）阴性的45例东亚肺腺癌患者中，有17例患者（37.8%）为MET 14外显子跳跃突变^[13]^。Cortot等^[14]^认为，MET 14外显子跳跃突变不与其他驱动突变共存，提示其代表了一种独立的致癌驱动基因。

目前临床病理特征的研究显示，MET 14外显子跳跃突变多发生于NSCLC，其中以肺肉瘤样癌和腺癌最为多见。在全基因组分析的38, 028例晚期恶性肿瘤患者中，共筛选出224例MET 14外显子跳跃突变的患者（总发生率约0.6%），发生率较高的肿瘤类型依次为肺腺癌（3%, 131/4, 402）、其他肺肿瘤（2.3%, 62/2, 669）、脑胶质瘤（0.4%）^[15]^。MET 14外显子跳跃突变在NSCLC中的总发生率为3%-6%^[11, 16]^，在肺腺癌中的发生率为3%-4%^[10, 11]^，在肺肉瘤样癌中的发生率可高达22%（8/36）^[17]^。而在中国人群中，Liu等^[18]^分析了968例中国NSCLC患者的DNA，发现仅有12例患者具有MET 14外显子跳跃突变，占腺癌患者的0.9%，明显低于高加索人群的发病率。

MET 14外显子跳跃突变人群年龄较大并且多有吸烟史^[10, 13, 19]^。Awad等^[10]^报道的28例MET 14外显子跳跃突变患者中位年龄为72.5岁，68%为女性以及64%为吸烟者。较*EGFR*突变患者（中位年龄61岁）及*KRAS*突变患者（中位年龄65岁），MET 14外显子跳跃突变患者年龄更大（*P* < 0.001）。Award等^[10]^还发现MET 14外显子跳跃突变的Ⅳ期患者更倾向于同时伴有*MET*基因扩增及MET蛋白过表达。但在一项更大样本量（共纳入298例MET 14外显子跳跃突变患者）的研究中，发现约20%患者同时伴有MET基因扩增，但与分期无关^[20]^。

MET 14外显子跳跃突变对NSCLC患者总生存的影响尚存在争论。在一项纳入了687例手术切除的NSCLC标本的研究中，多因素分析结果显示MET 14外显子跳跃突变是一项独立的预后不良指标^[12]^。然而Gow等^[11]^针对850例东亚NSCLC人群的研究表明，MET 14外显子跳跃突变患者的总生存与驱动基因突变阴性的患者并没有差异。

## 3 MET 14外显子跳跃突变患者的治疗策略

靶向MET的酪氨酸激酶抑制剂（tyrosine kinase inhibitor, TKI）为选择性的三磷酸腺苷（adenosine-triphosphate, ATP）竞争性抑制剂，通过抑制MET的活性进而阻止下游相关激酶的磷酸化，使下游信号转导停滞，以此调控肿瘤细胞的增殖、转移及血管形成。许多临床个案报道MET 14外显子跳跃突变患者经TKI治疗取得了客观缓解，见表 2^[10, 15, 17, 21-27]^。Heist等^[16]^建议，所有肺癌患者均应进行MET 14外显子跳跃突变检测，因其是一个全新的癌基因靶点，可作为靶向治疗的良好指标。但对这些数据的解读仍存在样本量小、有选择偏倚等问题，MET抑制剂对MET 14外显子跳跃突变患者的疗效仍需大样本量的临床研究来证实。

**2:** MET 14外显子跳跃突变患者的个案报道 Case reports of MET exon 14 skipping alterations

Year	Age	Sex	Smoking history	Histology	MET ex14 alteration	MET IHC	MET amplification	Agent	Best response	Reference
2015	61	M	No	Sarcoma	Splice site mutation	NA	NA	Crizotinib	PR	Lee, *et al*^[21]^
2015	68	F	Yes	Adenocar-cinoma	Splice donor mutation	NA	NA	Crizotinib	PR	Jorge, *et al*^[22]^
2015	66	F	Yes	Squamous	Splice donor mutation	3+	Yes	Capmatinib	PR	Frampton, *et al*^[15]^
2015	82	F	Yes	Large cell	Splice donor mutation	3+	NA	Capmatinib	PR	Frampton, *et al*^[15]^
2015	86	M	No	Adenocar-cinoma	Splice acceptor mutation	2+	NA	Crizotinib	PR	Jenkins, e*t al*^[23]^
2015	71	M	Yes	Adenocar-cinoma	Splice donor D1028H mutation	NA	No	Crizotinib	PR	Waqar, *et al*^[24]^
2015	76	F	Yes	Squamous	Splice donor D1010H mutation	NA	NA	Crizotinib	PR	Mendenhall, *et al*^[25]^
2015	80	F	No	Adenocar-cinoma	Splice donor mutation	Overexpr-ession^*^	Yes	Cabozantinib	SD	Paik, *et al*^[26]^
2015	78	M	Yes	Adenocar-cinoma	Splice donor deletion	Overexpr-ession^*^	NA	Crizotinib	PR	Paike, *et al*^[26]^
2015	65	M	Yes	Adenocar-cinoma	Splice donor mutation	NA	NA	Crizotinib	PR	Paik, *et al*^[26]^
2015	90	F	No	Adenocar-cinoma	Splice donor mutation	NA	NA	Crizotinib	PR	Paik, *et al*^[26]^
2016	67	F	No	Adenocar-cinoma	Splice donor D1028N mutation	NA	NA	Crizotinib	PR	Mahjoubi, *et al*^[27]^
2016	64	F	No	Adenocar-cinoma	Splice donor mutation	Overexpr-ession^*^	Yes	Crizotinib	PR	Award, *et al*^[10]^
2016	74	F	Yes	Sarcoma	Splice site mutation	NA	Yes	Crizotinib	PR	Liu, *et al*^[17]^
IHC: immunohistochemistry; M: male; F: female; NA: not applicable; PR: partial response; PD: progressive disease; ^*^H score=300.										

在这些抑制剂中，克唑替尼有望第一个获准治疗MET 14外显子跳跃突变患者^[28, 29]^。Profile 1001研究入组了21例MET 14外显子突变的晚期NSCLC患者，其中16例为腺癌，中位年龄为68岁，71%为女性。经过克唑替尼（250 mg *bid*）治疗，部分缓解率为44%，疾病稳定率为50%，未出现疾病进展。中位随访时间5.7个月，无进展生存期（progression free survival, PFS）和生存期（overall survial, OS）尚不成熟^[30]^。目前，克唑替尼用于治疗MET 14外显子跳跃突变患者的Ⅱ期临床试验正在进行，包括美国的NCI-MATCH trial（NCT02465060）和英国的National Lung Matrix Trial（NCT02664935）。另外，临床研究也证实了MET 14外显子跳跃突变的肉瘤样癌对克唑替尼可取得显著的治疗效果^[21]^。

## 4 MET 14外显子跳跃突变患者耐药机制及应对

由于基因扩增、第二位点突变、旁路激活以及病理类型转变等情况的存在，靶向治疗不可避免的一大问题就是耐药。MET-TKI分为Ⅰ型和Ⅱ型。Ⅰ型TKI（克唑替尼、Capmatinib、Ensartinib和沃利替尼）结合在ATP位点，与MET主链中的氨基酸残基形成氢键。体外研究表明，MET活化域，尤其是与Ⅰ型抑制剂相互作用的活化环发生突变是Ⅰ型抑制剂产生获得性耐药的主要原因。这些突变主要包括MET 14外显子Y1230突变和MET 19外显子D1228突变，可以直接或间接地减弱Ⅰ型抑制剂与MET活化环之间的结合。Ou等^[31]^报道了1例初治时MET 14外显子D1010H突变（丰度44%）伴有低丰度MET 14外显子Y1230C（0.3%）的NSCLC患者，克唑替尼治疗后疾病缓解持续13个月，疾病进展时二次检测发现D1010H突变丰度仅为11%，而Y1230突变丰度达3%。而另1例MET 14外显子D1010H突变的肺鳞癌伴有多发骨转移和肝转移患者，接受克唑替尼治疗后，疾病部分缓解达8个月。后续出现新的肝转移灶，取肝脏新生结节活检经检测为*MET*基因二次突变（D1228突变）^[32]^。由于作用机制的不同，Ⅱ型抑制剂（如卡博替尼，Glesatinib等）或许可以逆转由Y1230等突变引起的Ⅰ型抑制剂耐药^[33, 34]^。Ⅱ型TKI一般为多靶点酪氨酸激酶抑制剂，不仅占据ATP结合位点，还能通过管家基因突变进入非活性DFG-out构象形成的疏水口袋，对产生二次突变的MET仍具有抑制作用^[35]^。Bahcall等^[36]^报道有*EGFR*突变患者在第三代EGFR-TKI Osimertinib联合Ⅰ型MET-TKI沃利替尼治疗进展后，出现*MET* D1228V的基因突变并且丰度持续增加。该患者的治疗方案变成Erlotinib（100 mg/d）联合卡博替尼（60 mg/d）的治疗，很快症状改善，疾病缓解时间持续5个月以上。Klempner等^[37]^报道了1例65岁男性MET 14外显子跳跃突变伴多发肝转移和脑转移的NSCLC患者，服用克唑替尼4周后肺部肿瘤缓解，但多发脑转移，同时肝转氨酶升高至4级。换用卡博替尼60 mg/d，4周后脑转移完全消失，肺部肿瘤持续缩小，肝转氨酶恢复到正常。以上的研究均显示Ⅱ型MET-TKI或许可以克服Ⅰ型的耐药。

## 5 结语

MET 14外显子的跳跃突变显示了一种临床独特的NSCLC分子亚型，NCCN指南推荐了克唑替尼的治疗。目前仍有一些问题没有解决：首先，中国的MET 14外显子突变的NSCLC比例似乎低于高加索人群，需要进一步分析流调研究中检测方法和MET的探针序列。其次，*MET*基因具有异质性，临床上发现有*MET*突变和扩增及蛋白过表达共存的NSCLC患者，经过克唑替尼治疗有效，但尚未确定哪一种因素发挥了主要的肿瘤驱动作用; 此外，Ⅱ型MET抑制剂耐药的机制和治疗策略还不清楚，需要进行重复活检和全基因测序，分析可能的耐药机制进行个体化的治疗。总之，在精准医学的时代，分子检测非常重要。随着对肿瘤驱动因素和耐药机制的研究深入，MET 14外显子跳跃突变的NSCLC患者将会获得更好的疗效和长期的生存。
